# In-patient Tolvaptan use in SIADH: care audit, therapy observation and outcome analysis

**DOI:** 10.1186/s12902-017-0214-2

**Published:** 2017-11-06

**Authors:** Malik Asif Humayun, Iain C. Cranston

**Affiliations:** 1 Department of Endocrinology & Diabetes, Milton Keynes University Hospital NHS Foundation Trust, Milton Keynes, MK6 5LD UK; 2 Department of Endocrinology & Diabetes, Queen Alexandra Hospital Portsmouth, Portsmouth, PO6 3LY UK

## Abstract

**Background:**

Indications for use of tolvaptan in SIADH-associated hyponatraemia remain controversial. We audited our local guidelines for Tolvaptan use in this situation to review treatment implications including drug safety, hospital admission episode analysis (episodes of liver toxicity, CNS myelinolysis, sodium-related re-admission rates), morbidity; mortality and underlying aetiologies.

**Methods:**

We report a retrospective case series analysis of on-going treatment outcomes (case-note review) for 31 patients (age 73.3 ± 10.5 years, 55% females) consecutively treated with Tolvaptan as in-patient for confirmed SIADH with persistent S/Na^+^ < 125 mmol/L despite removal of reversible causes and 24-48 h fluid restriction, and include longer-term outcome data (re-treatment/readmissions/mortality) for up to 4 years of follow-up. A minimum of 6 months follow-up data were reviewed unless the patient died before that period.

**Results:**

Short-term outcomes were favourable; 94%-achieved treatment targets after a mean of 3.48 ± 2.46 days. There was statistically significant rise in S/Na^+^ level after Tolvaptan treatment (before treatment: mean sodium 117.8 ± 3.73, 108–121 mmol/L and after treatment: mean sodium 128.7 ± 3.67, 125–135.2 mmol/L, *P* < .001). Although the target S/Na^+^ level was >125 mmol/L in fact one third (35%) of the patients achieved a S/Na^+^ level of >130 mmol/L by the time of hospital discharge. No patient experienced S/Na^+^ rise >12 mmol/L/24 h, drug-associated liver injury or CNS-myelinolysis. The average length of hospital stay following start of Tolvaptan treatment was 3.2 days. Relapse of hyponatraemia occurred in 26% of the patients, requiring retreatment with Tolvaptan. In all patients where either relapse of hyponatraemia occurred or readmission was necessary, SIADH was associated with malignancy, which was present overall in 60% of the group studied.

**Conclusions:**

This study confirms the safety and efficacy of Tolvaptan in the treatment of SIADH-related significant, symptomatic hyponatraemia when used under specialist guidance and strict monitoring. A sodium level relapsing below the treatment threshold by 1 week after discontinuation is a good indicator of a patient group with re-treatment/longer-term therapy needs, all of whom had underlying malignancy. The criteria set locally in our trust to initiate Tolvaptan use also identifies a group where further investigation for underlying malignancy should be considered.

## Background

Hyponatraemia is the most commonly recorded electrolyte abnormality occurring in 7% to 8% of elderly, ambulatory patients and 15 to 20% of hospitalized patients [1–4] presenting with a variety of symptoms ranging from very mild to life threatening (e.g. from dizziness/nausea to seizures and coma). The treatment of SIADH needs to take into account the duration of the hyponatraemia and the degree of symptoms relating to it. Correction of S/Na^+^ has been shown to improve the symptoms and signs associated with this condition, although the impact on longer-term outcomes and survival benefits remain a topic of debate. It is also recognised that excessively rapid correction of S/Na^+^ can be detrimental [5].

For many patients affected acutely by hyponatraemia, the biochemical level is relatively mild and transient, and without symptoms reflecting significant fluid balance abnormalities [5]. However, even mild degree of persistent hyponatraemia has been associated with significant morbidity including impaired gait stability and increased falls and osteoporosis [6–8]. Therefore, prevention and treatment should be a clinical priority in patients with chronic hyponatraemia. During hospital admission, hyponatraemia is also associated with increased length of stay and worse primary clinical outcomes, generating a rationale for effective correction during the admission although this has yet to be associated with improved long term outcomes.

Acutely, fluid restriction remains the mainstay of treatment for moderate hyponatraemia associated with SIADH despite a relatively limited evidence-base, as historically the treatment of SIADH has been largely driven by expert opinion [9]. However, fluid restriction is often challenging and unpleasant for patients and when therapies such as chemotherapy for malignant disease are planned it is not a preferred option due to risk of dehydration and acute kidney injury. Patients, who are intolerant of, or have a poor response to fluid restriction after 24–48 h, are thus considered a priority for the use of pharmacological interventions. Many pharmacological treatments including demeclocyline, lithium, loop diuretics in combination with sodium supplementation and urea tablets have been trialled over the years in addition to fluid restriction but without consensus being reached on the safest and most effective strategy [10]. To this uncertain arena, the AVP receptor antagonist offers a potentially attractive therapy option as all episodes of SIADH involve dysregulation of AVP or its receptor to some degree.

Tolvaptan is the only UK-licenced vasopressin receptor antagonist; it has a greater affinity and selectivity for the V2 receptor than endogenous AVP [11]. Antagonism at the V2 receptor causes a decrease in the number of aquaporin-2 channels in the renal collecting tubules, resulting in decreased water reabsorption, a net increase in free water excretion (aquaresis), and an increase in serum sodium concentrations. This decrease in free water is not associated with an increased excretion of sodium or potassium ions; the increase in serum sodium concentration is solely a result of aquaresis [11]. Various studies have investigated the usefulness of V2 receptor blockers showing that serum sodium can be safely improved in patients with hyponatraemia with some additional benefits in patients’ quality of life, as measured by 12-item Short Form general health survey scores [2].

**Fig. 1:**
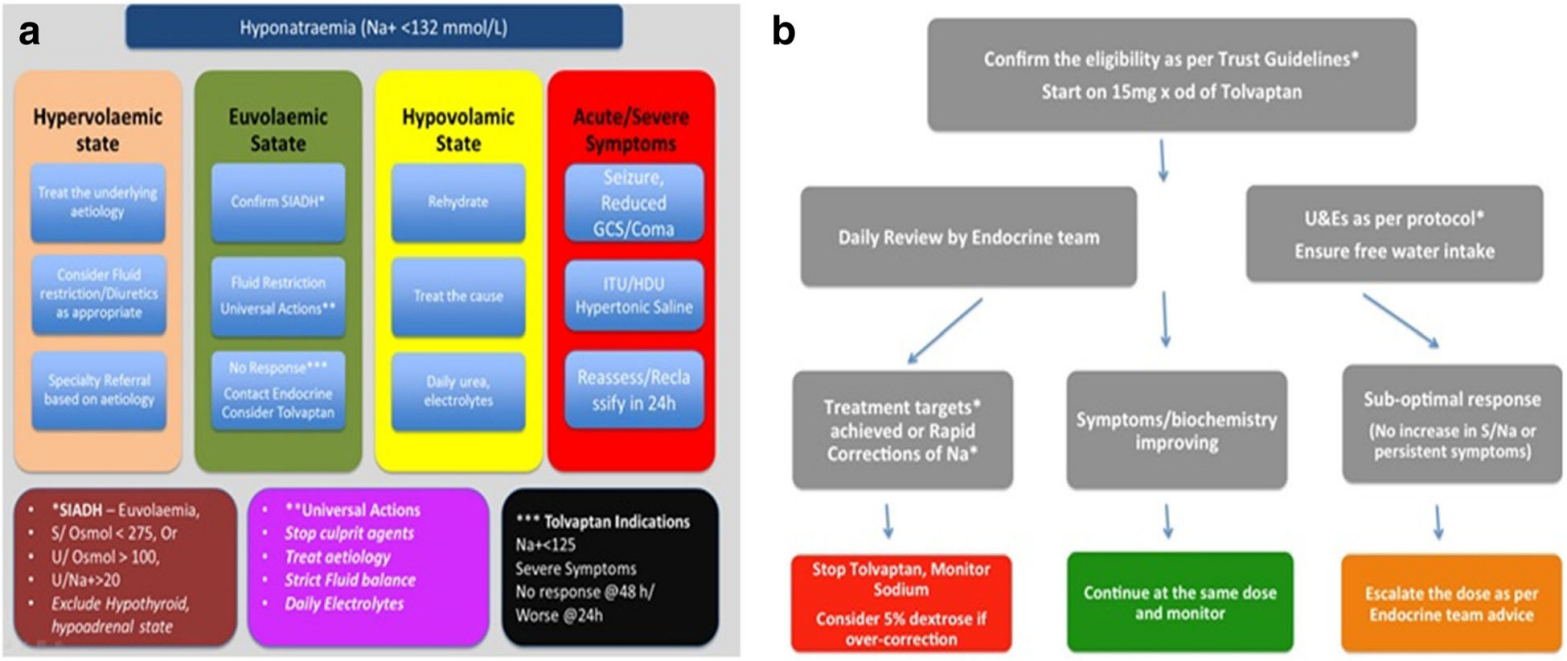
**a** Hyponatraemia Pathway S/Osmol, Serum Osmolality(mOsmol/kg); U/Osmol, Urine Osmolality(mOsmol/kg); Na+, Sodium (mmol/L); SIADH, Syndrome of Inappropriate ADH; GCS, Glasgow Coma Scale. **b** Tolvaptan Monitoring Policy. U&Es, Urea and Electrolytes. *Rapid correction of Na + is defined as rise in S/Na+ > target rise of 6 mmol/L over 12 h (and not exceeding 12 mmol/L over 1st 24 h) and an additional 8 mmol/L during every 24 h thereafter until the serum sodium concentration reaches 130 mmol/L.

In 2011, Tolvaptan was approved for limited use by our local trust formulary committee to treat hyponatraemia secondary to SIADH, which is not responsive to 24–48 h fluid restriction. The committee took a cautious approach to the approval of the drug because of controversy surrounding the outcomes measurement of acute hyponatraemia management. The diagnostic criteria and the protocol to use Tolvaptan as approved locally are shown in Fig. 1a and Table 1a respectively and this treatment was initiated and monitored only by the Endocrine team. Any patient clinically assessed to have severe hyponatraemia was transferred to a high care area for initial management with hypertonic saline prior to a decision regarding on-going management (Fig. 1a – Hyponatraemia Pathway).

**Table 1:** Indications for Tolvaptan Initiation

Tolvaptan – Initial usage policy
ᅟ1) Confirm SIADH (See Fig. 1a) AND
ᅟ2) Na <125 despite 48 h fluid restriction at 750 ml/24 h or
ᅟ3) Na <125 and falling despite 24 h fluid restriction @ 750 ml/24 h or
ᅟ4) Symptoms/risks associated with 2) or 3) awaiting for Chemotherapy
At initiation of Tolvaptan
ᅟ1) Stop fluid restriction (allow patient to drink when thirsty)
ᅟ1) Monitor Na levels 6hrly for 36 h then 12hrly
ᅟ1) Review Tolvaptan use at 3, 6 and 9 days
ᅟ1) If Na rises by >6 at 6 h, >8 mmol/L in 12 h, >12 mmol/L in 24 h or >18 in 48 h OR if Na >126, STOP Tolvaptan and continue to monitor Na 12hrly for 36 h – Consider 5% Dextrose.

➣ *Ensure free water intake. Avoid Tolvaptan if oral intake is inadequate/unsafe*

➣ *Ensure details are kept for each patient in which Tolvaptan is used according to attached proforma*

➣ *Where SIADH is associated with recurrent severe hyponatraemia Na <125 there may be a case to extend prescription to the outpatient setting, but this should be made as an individual case to the F&M chair*

An initial short-term audit was done by our department after 1 year of guideline use [12] to see the efficacy of Tolvaptan in patients with hyponatraemia secondary to SIADH and Tolvaptan was found to be safe and efficacious in the management of hospitalised in-patients with hyponatraemia, when initiated and monitored in the hospital setting under specialist supervision and following appropriate guidelines. It was also helpful in expediting the discharge of those patients who were admitted to the hospital mainly due to their hyponatraemia.

In 2014 we re-audited our practice in the management of hospitalised patients with significant SIADH related hyponatraemia in light of alerts over potential long-term side effects of this drug. Our project also aimed to review the level of concordance with specialty guidelines and if there was evidence to suggest the guidelines should be modified.

Additionally with over 4 years of experience in the use of Tolvaptan we wished to review the longer-term effect of hyponatraemia and treatment implications. We also included the study of drug safety and hospital episode analysis (episodes of liver toxicity, CNS myelinolysis, and sodium-related re-admission rates), morbidity; mortality and underlying aetiologies, in order to further inform our clinical practice.

## Methods and data analysis

This was a retrospective consecutive clinical case series study of tolvaptan use administered according to locally agreed clinical guidance (Nov 2010) targeting use for in-patients with severe SIADH-associated hyponatraemia. Who had not responded to traditional measures. The guidelines were deliberately cautious in the use of the drug, ensuring that it was removed once a threshold level of 126 had been achieved. Clinical notes and drug charts of all the patients treated with Tolvaptan (minimum 1 dose), from January 2011 to December 2014 and with a minimum of 6 months follow-up data were reviewed unless the patient died before that period.

All patients prescribed Tolvaptan during their admission were recorded on the pharmacy stock control and dispensing system (JAC version 4.43), and 34 patients were identified from their records. It was not possible to track the clinical record of two patients so data from 32 patients was analysed. Diagnostic criteria for SIADH were not met in one of the patients (A very symptomatic patient with previous history of SIADH, who was started on Tolvaptan without confirmatory tests) so 31 were included in the study. All the patients included in our study were trialled on fluid restriction and in some cases were given hypertonic saline or demeclocycline as appropriate. All patients with acute severe hyponatraemia were treated with hypertonic saline and managed on level 2 or 3 care facilities if suitable for escalation to intensive care settings. Tolvaptan was only used in patients who did not respond to the other interventions and had a normal fluid intake with GCS > 13 to avoid rapid correction of sodium. All the patients were started on 15 mg once daily of Tolvaptan initially.

Clinical data of all the patients included in our audit were collected from case notes, pathology computer systems (Sunquest ICE), and patient observation tracking system (VitalPAC) and patient letters management system (Graphnet). Information collected included demographic profile, clinical and biochemical parameters of the diagnostic criteria to establish the diagnosis of SIADH including drug history, documentation of fluid status, paired serum and urine osmolalities, Thyroid function tests and 9 am cortisol. The underlying aetiology of SIADH was documented wherever available. Other parameters regarding the use of Tolvaptan included the serial S/Na^+^ levels at 0, 6, 12, 24, 48, 72, 96 h after the treatment with Tolvaptan, total duration of Tolvaptan treatment and if the patients required any retreatment. The success of treatment was defined as the improvement of S/Na^+^ above a threshold of 125 meq/L and documented improvement in the clinical features, which might be related with hyponatraemia. All the patients successfully treated with Tolvaptan had their S/Na^+^ levels monitored on alternate days following the discontinuation of treatment and if their sodium levels were >132 after 1 week their monitoring frequency was reduced to once monthly for minimum of 3 months. Patients who relapsed during the follow-up period were re-treated with Tolvaptan using the same monitoring protocol. All patients were informed that during tolvaptan treatment they should not restrict fluid intake and so were given free access to drinking water, taken according to thirst.

Compliance with local guidelines (Fig. 1, Table 1) and also the efficacy of Tolvaptan (improvement of serum sodium, expediting the discharge and prevention of re-admission at 1, 4, 12 and 24 weeks after the discharge) was also reviewed. Any incident of excessively rapid correction of hyponatraemia, central pontine myelinolysis or liver injury related with Tolvaptan was also documented.

This information was analysed on an Excel spread sheet with statistical analysis using Graphpad (http://www.graphpad.com). Continuous variables were expressed as means ± standard deviations or medians with interquartile ranges and tested by the Student’s t-test. Categorical variables are described as proportions and compared using the Chi-Squred test. The study was approved as an audit by the Queen Alexandra Hospital Portsmouth, NHS Trust (audit reference number 2590).

## Results

**Fig. 2:**
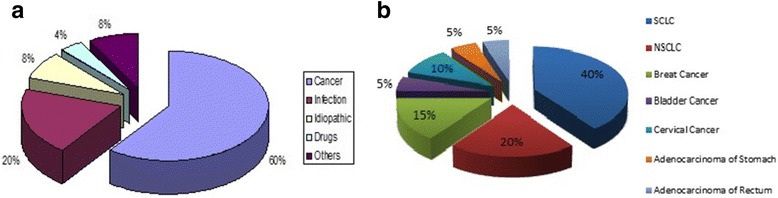
**a** Aetiology of SIADH. **b** Primary Sites for Underlying Malignancies Associated with SIADH. *NSCLC – Non-small Cell Lung Cancer, SCLC – Small Cell Lung Cancer.

**Fig. 3:**
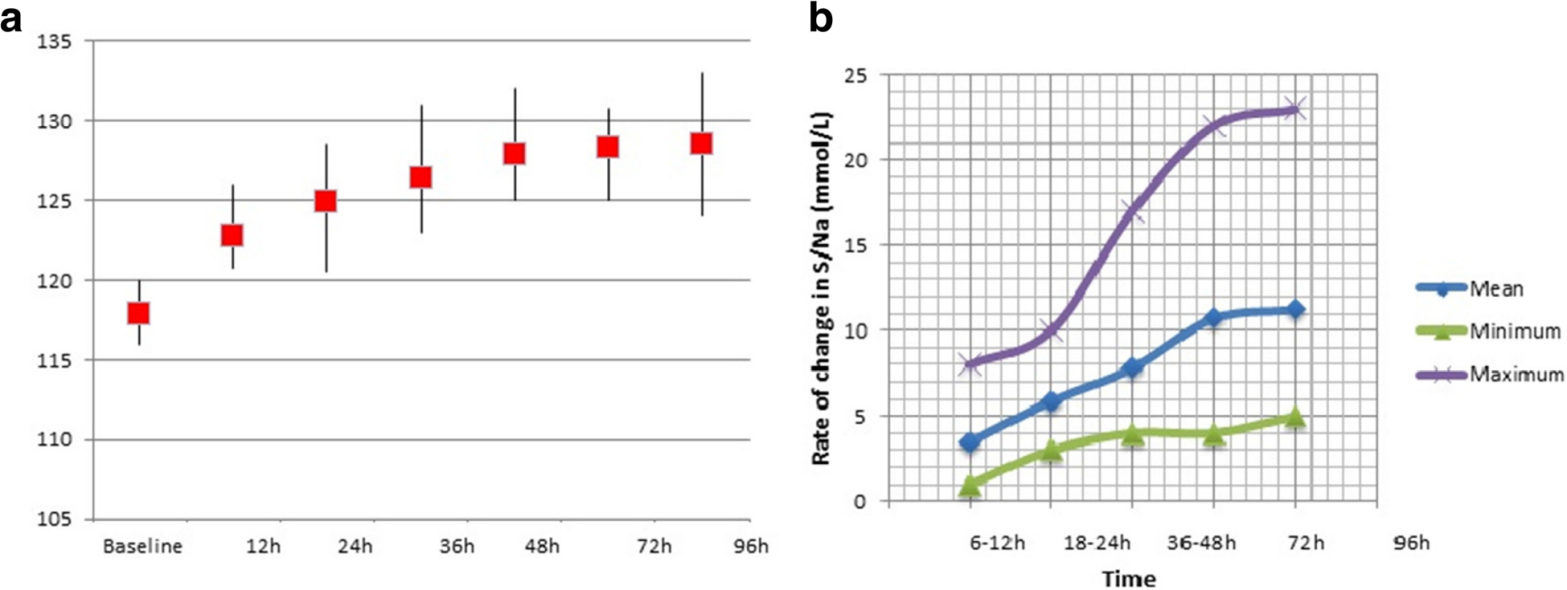
**a** Improvement in Sodium levels after Tolvaptan Initiation (Median, Interquartile Range). **b** Rate of change of Sodium from Baseline after start of Tolvaptan

There were 17 (55%) female and 14 (45%) male (mean age + standard deviation 73.3 +/− 10.5). Most common aetiology of SIADH was malignancy (Fig. 2a, b). Our observation showed good compliance with the local guidelines (Table 2). Treatment was successful in 94% of the case while two patients had sub-optimal response where the clinical symptoms were improved but S/Na^+^ levels did not rise above 125 mmol/L. The rate of change of S/Na^+^ after initiation of Tolvaptan treatment is shown in Fig. 3a and b. There was a significant rise in S/Na^+^ level after Tolvaptan treatment (before treatment: mean sodium 117.8 ± 3.73, 108–121 mmol/L and after treatment: mean sodium 128.7 ± 3.67, 125–135.2 mmol/L, *P* < .001). All of the treated patients had improvement in their hyponatraemia related symptoms (Table 3). Increase in S/Na^+^ was aimed to be <12 mmol/L/24 across the study population. Only one patient required 5% dextrose infusion when increase in sodium at 12 h was 11 mmol/L. The mean duration of treatment was 3.48 days (Std. Deviation 2.46). Side effects observed with Tolvaptan are shown in Table 4.

**Table 2:** Adherence with local guidelines

Diagnosis confirmed clinically and biochemically	100%
Removal of potential culprit drugs	100%
Exclusion of adrenal / thyroid pathology	100%
Documentation of fluid balance status and charts	92%
Documentation of 48 h fluid restriction prior to starting Tolvaptan	96%
Tolvaptan initiation according to Trust policy	96%
Na level monitoring before/after initiation as per recommendations	96%

**Table 3:** Common symptoms before and after Tolvaptan treatment

Symptoms	Before Treatment	After Treatment
Drowsiness	38%	0%
Confusion	19%	0%
Fatigue	58%	6.4%
Miscellaneous/Nonspecific	28%	9.6%

**Table 4:** Common Side effects of Tolvaptan treatment

Dry Mouth	22%
Thirst	18%
Polyuria	5%
CNS myelinolysis	0%
Elevation of Liver Enzymes	0%

Following discontinuation of Tolvaptan, S/Na^+^ remained above the treatment threshold of 126 mmol/L in 74% of the patients. Relapse of hyponatraemia below 126 mmol/L following therapy discontinuation occurred in 26% of the patients. All of the patients whose S/Na^+^ relapsed below 126 mmol/L did so within 1 week after stopping the Tolvaptan treatment. This group was re-treated successfully with Tolvaptan, however further investigations revealed that the entire group requiring re-treatment had an underlying malignancy and required longer-term Tolvaptan treatment (9 days to 12 weeks). Three of these patients were able to receive re-treatment with Tolvaptan as an outpatient. One of these patients died after 2 months while other two managed to come off their Tolvaptan treatment following a good response to their cancer chemotherapy.

Majority (60%) of the patients did not require any further increase in the dose of Tolvaptan as per protocol. In rest of the 40% patients dose was gradually increased by 15 mg every 2–3 days depending upon response. Only two patients required doses higher than 30 mg/day. The average length of hospital stay following the start of Tolvaptan treatment was 3.2 days.

Further analysis of the patients with underlying malignancy showed that 47% (9/19) received chemotherapy and 66% (6/9) were able to come off Tolvaptan completely with in 2 weeks while three patients required longer-term Tolvaptan. Hyponatraemia-related re-admission was observed in 13% of the patients after stopping their Tolvaptan treatment prior to hospital discharge. These patients presented with in a week with confusion and falls but none of the patients was reported to have seizure activity or significantly reduced GCS as a presenting feature. Again, all these patients had an underlying malignancy and were re-treated successfully with Tolvaptan. There was no relapse of hyponatraemia or re-admission in patients who did not have an underlying malignancy (40% of study population). In this group of patients there was no deaths over the study period (minimum 6 months) except for those with underlying neurological disease both of whom died within a month.

**Fig. 4:**
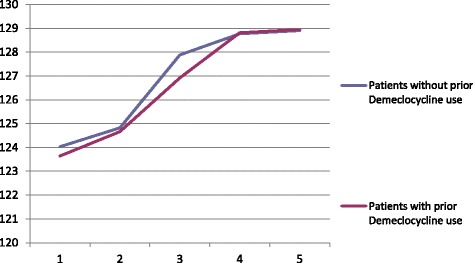
Comparison of Response to Treatment Between Patients with and without Prior Use of Demeclocycline

About one third (32%) of the patients initially treated had unsuccessfully trialled fluid restriction with demeclocycline for at least 4 days (maximum 10 days) prior to starting Tolvaptan treatment. There was no difference in the response to Tolvaptan treatment between patients with prior use of demeclocycline to the ones without previous use of demeclocycline (Fig 4).

A significant proportion (60%) had underlying malignancy of which 90% were undiagnosed at the time of their presentation. This group of the patients showed a very poor prognosis with 46% mortality over 3 months and 75% over the follow-up period. The prognosis was relatively good in the patients without having an underlying malignancy. Mortality was 16% (2/12) in this group and both these patients had underlying severe neurological pathology (Sub-Arachanoid Haemorrhage and Cerebral Infarction).

## Discussion

This study confirms the safety and efficacy of Tolvaptan in the treatment of SIADH-related hyponatraemia when used in carefully selected group of patients under specialist guidance and strict monitoring.

The efficacy and safety results of Tolvaptan in the treatment of SIADH in our study are consistent and comparable to several studies [2, 13–15]. However despite all available evidence, the use of Tolvaptan remains rather limited in daily clinical practice. The use of Tolvaptan in SIADH was noted to be in only 5% of the subjects in hyponatraemia registry [13].

One of the most important concerns limiting the use of Tolvaptan into daily clinical practice is the risk of rapid correction of sodium (Sodium rise >12 mmol/L in 24 h or 18 mmol/L over 48 h) and CM. The reported risk of over-correction of hyponatraemia in various studies varies significantly. Tzoulis et al. [15] recently publish a real life experience of Tolvaptan use in two hospital and noted a rapid rise of S/Na > 12 mmol/L in 18% of their patients while the rate of overcorrection was noted to be up to 11.7% (10.8% when used as montherapy) in hyponatraemia registry [9]. Verbalis et al. [14] reported that 5.9% of their study population showed over correction. This is in contrast to our patient population where a rise in S/Na > 12 mmol/L in 24 h was not noticed. This difference could be due to variety of reasons. The risk of rapid overcorrection of sodium is increased if patient develops dehydration due to reduced oral intake of water; concomitant use of diuretics or other pharmacotherapy potentiating the effect of Tolvaptan or if SIADH is improving after successful treatment of its underlying aetiology like infection etc. Similarly pituitary surgery is a situation where SIADH may be transient and resolve suddenly [13]. The clinical characteristics of the patients experiencing rapid overcorrection in the study by Tzoulis et al. [15] are not described in detail but one of participating hospital is a major neurosurgical centre and over 20% of their study population had an underlying neurological cause or were post-operative following a neurosurgical procedure. Only 24.6% in their study population had malignancy as an underlying aetiology of SIADH in contrast to our study population where 60% of the patients had an underlying active malignant process and only 6.4% had a neurological cause and none of the patients had pituitary/neuro surgery and all of our study patients had a normal Glasgow Coma Scale with free access to oral fluids and all interfering medications were stopped at the time of diagnosis of SIADH.

Higher proportion of malignancy in our group could explain a rather gradual rise in sodium as patients with SIADH secondary to underlying malignancy have a more severe and resistant hyponatraemia compared to other aetiologies of SIADH as in these patients, SIADH may not only be a result of ectopic AVP production by tumor cells but can also be a result of stimulation of AVP secretion or potentiation of AVP effects by anticancer drugs or palliative medications [16]. Moreover, our local policy for the dose escalation of Tolvaptan (Fig. 1b) is less aggressive, study population included patients with severe hyponatraemia, resistant to usual treatment. All these factors and aggressive monitoring of sodium might have contributed to the less rapid rise in sodium and avoidance of the complications.

None of our patients exhibited any neurological symptoms suggestive of osmotic demyelination, which has been true for all previously reported studies of vasopressin receptor antagonists [2, 14, 15]. The only case of CNS myelinosis with the use of a vasopressin-receptor antagonist, reported by Malhotra et al. [17] is a typical example where a fluid overload state was treated with Tolvaptan at day six of hospital admission and aggressive diuretic treatment was given before that. It is unclear whether patient remained on the diuretic therapy during Tolvaptan treatment or not. Unfortunately, despite achieving targets/Na, Tolvaptan was not discontinued and was rather increased to 30 mg/day, which led to a massive rise in S/Na to 187 mmol/L leading to osmotic demyelination.

Nevertheless, due to extremely poor outcomes associated with CNS myelinolysis, treating physicians should be vigilant about the symptoms suggestive of this complication when using Tolvaptan to correct patients with SIADH. Careful selection of the patients who could benefit from this treatment, rigorous sodium monitoring and prompt intervention including therapy discontinuation soon as target sodium is achieved appear to be the best strategies to avoid rapid over-correction of sodium and development of CNS osmotic demyelination.

The other real-life concern, limiting the wider use of Tolvaptan, remains its cost. Several investigators have recently showed Tolvaptan to be a cost effective option for hospitalised patients with hyponatraemia secondary to SIADH in hospitalised patients due to reduced length of hospital stay and improvement in quality of life analysis [18–20]. Similar conclusions cant not be drawn based on our results due to limited sample size and absence of a control group but an average length of hospital stay following Tolvaptan treatment was 3.2 days which appears to be a success as majority o these patients were in-patients while being on fluid restriction and/or demeclocycline therapy for at least >48 h prior to the start of Tolvaptan treatment. This confirms the results from other studies that Tolvaptan is effective and safe therapy (when appropriately monitored and administered) and improves the symptomatic outcomes for patients without significant side-effect risks [21, 22]. Our study also highlights the importance of continued S/Na^+^ monitoring after therapy discontinuation, with an important predictive role for S/Na^+^ levels at 1 week as all the patients in relapse group relapsed with in a week after discontinuing their treatment.

To date, no clear guidelines or nationally or internationally accepted criteria exist for the use of Tolvaptan in SIADH. The recommendations for the use of Tolvaptan are at variance when comparing the American and European guidelines for the management of hyponatraemia. The American guideline [23] represents a consensus opinion by a panel of hyponatraemia experts. These experts recommended the use of Tolvaptan in the management of non-severe hyponatraemia due to SIADH, liver cirrhosis and heart failure when fluid restriction has failed as first line therapy. The European guidelines [24] do not support the use of Tolvaptan due to the lack of morbidity and mortality benefits as well as due to some concerns regarding their risk of liver injury and CNS myelinosis due to potential overcorrection of S/Na. These guidelines recommended against Tolvaptan use in hypervolaemic hyponatraemia. A panel of experts from UK [25] recommend the use of Tolvaptan in resistant hyponatraemia in their consensus guidelines.

All previous studies reporting use of Tolvaptan in real life do not clarify their local criteria for Tolvaptan use and monitoring. Some have admitted [14] that there was no clear local policy about the use of Tolvaptan in these patients. The use of Tolvaptan totally depends upon treating physician’s discretion and local policy. Therefore, it is not unlikely that sometimes patients with relatively mild hyponatraemia, which could normally be managed with fluid restriction, could be treated with Tolvaptan leading to unnecessarily rigorous sodium monitoring and significant cost of Tolvaptan treatment and vice versa. The selection criteria for Tolvaptan treatment reported in our study are robust and identify a group with severe hyponatraemia secondary to SIADH, resistant to first-line treatment and could potentially be considered for a consensus guidelines for the use of Tolvaptan. The clear monitoring guidelines following Tolvaptan treatment are also beneficial to the medical staff who are involved in the monitoring of patients treated with Tolvaptan during ‘out of hours’ and are helpful in the decision-making process.

It is also apparent that the protocol developed locally to identify patients for Tolvaptan treatment of their resistant hyponatraemia secondary to SIADH may also represent a predictor of potential underlying malignancy (60% predictive rate). This is particularly true for individuals who had a relapse of their SIADH following the discontinuation of initial Tolvaptan therapy (100% predictive rate). Many researchers believe that hyponatraemia should no longer be considered just a biochemical marker in critically ill patients [26] but whether malignancy related hyponatraemia is just a marker of poor prognosis or whether its prompt management may alter patient’s life expectancy or quality of life has not been properly studied. Balachandran et al. recently recently reported that correction of the sodium level leads to additional treatment and significantly greater overall survival [27]. However, from their study it was not possible to determine if this was an effect of specific therapy of the hyponatraemia or the resolving hyponatraemia reflected an association with improvement in the clinical condition. Nevertheless, treatment of hyponatraemia may allow more anti-cancer treatment options and help improve the survival. This series, undertaken as a safety review has highlighted the important clinical points but was not of adequate size, nor was it designed to determine if early primary diagnosis with aggressive investigation following a diagnosis of severe SIADH can impact on outcome. This question, and the level of diagnostic criteria, which indicate underlying malignancy risk needs to be further elucidated using other threshold values (e.g S/Na^+^ of <130 rather than 125 mmol/L) in a wider population group.

## Conclusions

Tolvaptan use remains a safe and effective in-patient short-term therapy option in carefully selected and monitored patient populations but its use in daily clinical practice is still limited due to the potential risk of overcorrections and its cost. There are no universally agreed criteria for the use of Tolvaptan in SIADH. The indications for the use of Tolvaptan in our local protocol include patients with severe/symptomatic hyponatraemia secondary to SIADH, who are resistant to the first-line therapy management with fluid restriction with or without prior use of demeclocycline and patients with SIADH having underlying malignancy, waiting for chemotherapy where the use of demeclocycline or fluid restriction is not desirable due to risk of dehydration and acute kidney injury following chemotherapy.

A sodium level relapsing below the treatment threshold by 1 week after discontinuation is a good indicator of a patient group with re-treatment/longer-term therapy needs, all of whom had underlying malignancy. Thus, the criteria set locally to initiate Tolvaptan use also identifies a group where further investigation for underlying malignancy should be considered.

## Abbreviations

AVP: Arginine Vasopressin
CNS: Central Nervous System
GCS: Glasgow Coma Scale
S/Na: Serum Sodium
SIADH: Syndrome of Inappropriate ADH secretion
